# Dolutegravir-based anti-retroviral therapy is effective and safe in HIV–infected paediatric patients

**DOI:** 10.1186/s13052-018-0469-x

**Published:** 2018-03-20

**Authors:** Eugenia Bruzzese, Andrea Lo Vecchio, Andrea Smarrazzo, Orsola Tambaro, Giulia Palmiero, Giovanni Bonadies, Alfredo Guarino

**Affiliations:** 1 Department of Translational Medical Science - Section of Pediatrics, University of Naples Federico II, Naples, Italy; 2 Unit of Viral Diseases, including AIDS DH, AOU Federico II of Naples, Naples, Italy

**Keywords:** HIV, Dolutegravir, Integrase strand transfer inhibitor, Adolescent, Children, Compliance, Side effects

## Abstract

**Background:**

Treatment of HIV infection in adolescents is challenging due to long duration of therapy and poor adherence. Recently, the integrase strand transfer inhibitor dolutegravir (DTG) has been approved for the use in adolescents with HIV, but evidence in clinical practice is very limited.

**Methods:**

We describe six cases of HIV-infected children/adolescents successfully treated with DTG-based regimen. Data relative to children/adolescents managed at the Referral Center for Pediatric HIV/AIDS of the University of Naples were reviewed. Patients were tested before introduction of DTG, after 1 month and every 3 months in the first 2 years to assess virologic and immunological response, tolerance and development of side effects. Families were asked to report any suspected adverse events.

**Results:**

Six patients (2 male, median age 17 years, range 12–18) were started on DTG-based anti-retroviral regimen due to low adherence to anti-retroviral treatment (ART), multiple drug resistance mutations, or development of ART-related side effects. Within 4–8 weeks after DTG treatment onset, a complete viral suppression and a concomitant increase of CD4^+^ cell count was observed. Four patients showed a persistent suppression after 2 years of follow-up, and 2 patients at about 1 year. One month after the introduction of DTG, the patient enrolled because of severe dyslipidaemia and hyper-transaminasemia showed a complete normalization of laboratory values. During follow-up (median 24 months, range 9–24) no adverse events were reported and most patients demonstrated a good adherence to treatment.

**Conclusions:**

DTG-based treatments demonstrated efficacy and good safety profile in adolescents. All patients demonstrated a rapid virologic and immunological response within 4–8 weeks, with good adherence and absence of side effects.

## Background

The treatment of adolescents with vertically-acquired HIV infection is particularly challenging [1] due to social and clinical factors. Poor adherence to long-term anti-retroviral therapy (ART), and consequent treatment failure with multiple therapy shifts, have been reported in adolescents with HIV infection. This condition could be related to multiple factors, including: the development of side-effects, the discovery of sexuality and acceptance of HIV-related “stigma”, the limited awareness of infection state and a general misinformation about HIV infection, often associated with socio-economically deprived communities [2, 3].

Several studies have identified both pill burden and lifestyle issues (i.e., not having medications on hand when away from home, changes in schedule) as major barriers to effective adherence. Choosing the simplest regimen possible, reducing dose frequency and pill number and selecting a regimen that fits well to daily patients and family activities, are important strategies to achieve.

Recently, the integrase strand transfer inhibitors (INSTIs) have been approved as preferred regimens for treating children and adolescents with HIV infection [4, 5]. Dolutegravir (DTG) has interesting pharmacokinetic/pharmacodynamic features such as prolonged intracellular half-life, absence of negative interactions with other anti-retroviral drugs and a potent activity against HIV-1 strains that are resistant to other INSTIs [6]. Its use is currently approved for treatment of HIV-infected adolescents (> 12 years old and/or above 40 kg of weight) [4, 5]. The approval was supported by data from a study of 23 treatment-experienced but INSTI-naive children and adolescents [7]. The drug has a very favourable safety profile and can be administered once a day to INSTI-naive patients [8]. These characteristics made DTG highly suitable for adolescents infected with HIV strains resistant to other anti-retroviral drugs.

Recently DTG, in combination with other anti-retroviral drugs, was effective in dramatically reducing viral replication without side effects in two patients, an adult [9] and a child [10], both with multidrug genetic resistance profile, and has been used to prevent mother-to-child HIV transmission [11].

## Methods

We performed a retrospective review of adolescents with HIV infection treated with DTG at the Regional Referral Center for Pediatric HIV of the University of Naples Federico II in collaboration with consultants from the Unit of Viral Diseases. Clinical and laboratory data were retrieved through manual chart reviewing. All patients aged ≤ 18 years old were tested before ART switch (T0), at 1 month (T1), and successively every 3 months after introduction of new ART regimen to assess virologic and immunological response and the potential development of side-effects.

Viral load was evaluated by using a real time-PCR (Abbott, with cut off sensitivity 40 copies/ml and dynamic range 40–1.000.000 copies/ml). Caregivers of children were required to report any suspect adverse event to the Reference Center after ART switch. Other potential side effects were specifically looked for during follow-up visits.

DTG was prescribed according to recent guidelines for the management of HIV infection in children and adolescent [4, 5].

No ethical committee opinion was requested because the use of this drug in adolescents is suggested by international guidelines. All procedures performed in this study were in accordance with the 1964 Helsinki declaration and its later amendments or comparable ethical standards. An informed consent for sharing anonymous patient data was obtained from families and/or caregivers. All drugs were provided by the University Hospital Pharmacy and paid by the National Health Care System according to Italian legislation. No direct contact with drug companies was taken.

## Results

### Dolutegravir-based therapy onset

Since January 2015 a DTG-based ART was prescribed to 6 paediatric patients (2 males, median age 17 years, range 12–18) due to previous treatment failure, development of side effects, presence of multiple viral resistance or low adherence to ART (Tables 1 and 2). An additional patient receiving a DTG-based regimen was excluded from the present study due to the lack of data after the first month of prescription (lost to follow-up).

**Table 1:** Description of the patients involved in the study

Patient	Gender	Age	Country of origin	CDC class	Previous ART regimen	New ART regimen	Reason for ART switch	Follow-up (months)
1	F	18	Italy	3	LPV/r + TDF/FTC	DTG + TDF/FTC	Side effects Low adherence	24
2	F	17	Nigeria	3	LPV/r + TDF/FTC	DTG + TDF/FTC	Side effects Low adherence	24
3	M	12	Italy	3	LPV/r + ABC + 3TC	DTG/ABC/3TC	Side effects	24
4	M	16	Italy	1	LPV/r + TDF/FTC	DTG + TDF/FTC	Side effects Low adherence	24
5	F	18	Nigeria	3	FPV + TDF/FTC + ATV + RTV	DTG + TDF/FTC	ART simplification	12
6	F	17	Ukraine	3	EFV + FTC + TDF	DTG/ABC/3TC	Side effects Low adherence	9

**Table 2:** Mutations and resistance pattern

Patient	Reverse transcriptase		Protease	
	Mutations	Resistance	Mutations	Resistance
1	M41 L, V75IMV, F77 L, T215D, E44A/D, V118I, D67N, M184 V	3TC (LR), ABC (IR), ZDV (HR), D4T (HR), DDI (HR), FTC (LR), TDF (IR), EFV (S), ETR (S), NVP (S), RPV (S)	A71AV, N88DN, D30N	LPV (S), ATV (LR), DRV (S), IDV (S), FPV (S), NFV (IR), SQV (LR), TPV (S)
2	K103 N, P225H, M184 V	3TC (HR), ABC (LR), ZDV (S), D4T (S), DDI (LR), FTC (HR), TDF (S), EFV (HR), ETR (S), NVP (HR), RPV (S)	K20I, A71AT, L89 V, M36I	LPV (S), ATV (S), DRV (S), IDV (S), FPV (S), NFV (LR), SQV (S), TPV (S)
3	Not available	Not available	Not available	Not available
4	M184 V, G190A	3TC (HR), ABC (S), ZDV (S), D4T (S), DDI (S), FTC (HR), TDF (S), EFV (IR), NVP (HR)	L10 V, L63P	LPV (S), ATV (S), DRV (S), IDV (S), FPV (S), NFV (S), SQV (S), TPV (S)
5	D67N, K101P, K103 N/S, M184 V, G190A, T215Y	3TC (HR), ABC (IR), ZDV (IR), D4T (IR), DDI (S), FTC (HR), TDF (S), EFV (HR), NVP (HR)	L10F, I13V, K20I, M36I, M46I, Q58E, L63P, H69K, L76 V, I84V, L89I	LPV (IR), ATV (HR), DRV (S), IDV (IR), FPV (HR), NFV (HR), SQV (IR), TPV (IR)
6	Not available	Not available	Not available	Not available

All but one patients were classified as stage 3 according to the 2014 Center for Disease Control and Prevention (CDC) Classification for paediatric HIV/AIDS [12] and none of them presented AIDS-defining conditions.

The clinical management of patient 1 and patient 2, both female in stage 3, was complicated by missed medical visits, a sustained low compliance to ART and the development of multiple genetic resistance mutations. They received various anti-retroviral drugs combined in different regimens: zidovudine (ZDV), stavudine (D4T), lamivudine (3TC), efavirenz (EFV), nelfinavir (NFV), lopinavir/ritonavir (LPV/r), tenofovir (TDF), emtricitabine (FTC).

The last ART regimen was LPV/r and TDF/FTC, which was poorly tolerated due to persistent gastrointestinal side effects in both patients. Particularly, although other alternative regimens including PI were considered, patient 1 experienced severe and frequent side effects related to PI, with recurrent abdominal and epigastric pain, nausea, episodes of vomiting and anorexia. Because of the persistence of virologic failure associated with a decrease of CD4+ cells, on January 2015, a once-daily regimen with DTG and TDF/FTC was started with the aim of increasing compliance and immunological and virologic response.

Patient 3 was a 12-year-old male admitted to our Department for acute pancreatitis whose mother died 8 years before due to AIDS-related meningitis, and he never underwent testing because of parental refusal. At the time of HIV diagnosis, he presented with 190 CD4+ cells/μl (8%) with a viral load of 19.800 copies/ml. Since the patient was naïve to anti-retroviral therapy, we started anti-retroviral treatment with LPV/r and ABC plus 3TC, according to Pediatric Guidelines for HIV infection [5]. After 4 weeks of treatment a good virologic response was obtained with a substantial reduction of HIV viral load (from 19.800 to 191 copies/ml, 2 logs in 4 weeks). However, after one month of LPV/r-based ART, the patient showed a sustained increase of total cholesterol (749 mg/dl), LDL cholesterol (513 mg/dl) and serum triglycerides (345 mg/dl), with concomitant reduction of HDL cholesterol (5 mg/dl). Dyslipidaemia was associated with hyper-transaminasemia (AST and ALT > 5 times normal values) and an increase in alkaline phosphatase (1774 mg/dl). Considering child age, the expected long duration of therapy and the elevated risk of persistent hypercholesterolemia and hyper-triglyceridemia associated to protease inhibitors-based regimens, the child was switched to a DTG-based regimen (DTG + ABC + 3TC) (Table 1).

Patient 4 was a 16-year-old Italian male who was on LPV/r + TDF/FTC in the last 4 years of follow-up, and subsequently developed chronic (not infectious) diarrhoea as side effect of ART. This was responsible for a progressive decrease in treatment adherence and he was switched to DTG-based regimen.

Patient 5 was an 18-year-old female treated with FPV + TDF/FTC + ATV + RTV due to a complex mutation profile. A DTG-based regimen was introduced in order to simplify the ART.

Patient 6 was a 17-year-old female from Ukraine treated with EFV + FTC + TDF. In order to improve compliance, a DTG-based regimen was started.

### Viro-immunological response

**Fig. 1:**
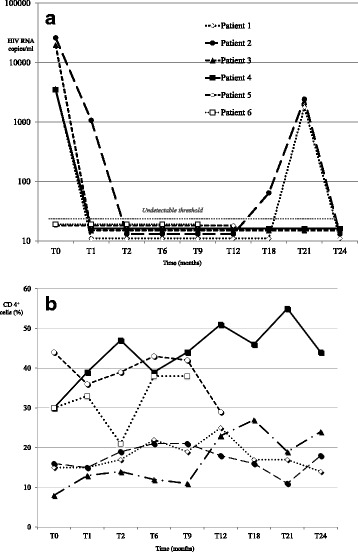
**a** Effects of dolutegravir-based regimen on viral load after the introduction of DTG-based regimen in 7 HIV-infected adolescents. **b** Effects of dolutegravir-based regimen on CD4+ cell percentage after the introduction of DTG-based regimen in 7 HIV-infected adolescents

Patients 1, 3 and 4 experienced full viral suppression (HIV viral load < 40 copies/ml) within the first 4 weeks. Patient 2 demonstrated a significative reduction (2 logs) after 4 weeks and a complete viral suppression after 8 weeks of treatment. Patients 5 and 6, who already had undetectable viral load under previous ART regimen, maintained a suppressed HIV load after treatment switch (Fig. 1 a).

During follow-up, two patients (1 and 2), discontinued ART and required unscheduled clinical visits that showed viral load peaks of 1779 copies/ml (patient 1) and 4100 copies/ml (patient 2). After discussions with both patients regarding the importance of adherence and the risks of acquiring further drug resistance mutations, we observed virologic suppression at subsequent visits, resulting from improved adherence to ART and rapid response to DTG-based ART.

A progressive increase in CD4+ cells was observed in all but one patients (Fig. 1 b).

Patient 3, who showed a major reduction in viral load during LPV/r-based regimen, demonstrated full viral suppression (HIV viral load < 40 copies/ml) and an increase in CD4+ cells after three weeks of DTG-based treatment (Fig. 1 a/b).

Patient 5, who was started on DTG-based regimen with a high percentage of CD4+, had a slight reduction of CD4+ at first control (from 44% to 36% with no significant reduction in absolute CD4+ lymphocyte count) and then remained substantially stable.

### Follow-up and safety

The 6 patients were followed during the last two years with a median follow-up of 24 months (range 9–24 months). All patients are still in follow-up at our institution and DTG-based treatment is still ongoing. Neither side effects or treatment failure were observed during follow-up visits. Two patients reported a transient reduction in ART adherence.

Patient 3, who received DTG-based regimen due to the presence of serious side effects, had a complete normalization of total (142 mg/dl) and LDL (80 mg/dl) cholesterol, triglycerides (74 mg/dl) concentrations and liver markers (AST 36 U/ml, ALT 36 U/ml, ALP 263 mg/dl) within one month after ART switch.

## Discussion

Despite excellent efficacy, safety and tolerability in adults, the effects of DTG in children and adolescents are poorly documented. DTG is approved for patients ≥ 12 years of age (recently anticipated at 6 years) and the IMPAACT study showed that 70% of adolescents (12 to < 18 years old) treated with DTG achieve a complete viral suppression [8]. Data from literature demonstrated that DTG is useful as a rescue drug in the setting of patients with multi-drug resistance [13].

In our experience, two patients who in the last five years never achieved a viral suppression with several ART regimens, showed a rapid and good response to DTG-based regimen. Both experienced a viral suppression and increase of CD4+ cells, although their adherence to ART was not optimal as demonstrated by a temporary viremia relapse. As reported in adult patients [14], due to its high genetic barrier, DTG may reduce the risk of developing viral resistance also in poorly compliant adolescents.

Patient 3, the only one receiving DTG-based regimen for serious ART-related side effects, reached a complete normalization of dyslipidaemia and hyper-transaminasemia, in association with a complete viral suppression, as previously reported in a large adult population [15]. The dramatic response of both viral load and CD4+ count, in association with the normalization of lipid profile, suggests that DTG-base regimen is a good therapeutic option also in naïve HIV-infected adolescents.

One limitation of this study is that drug resistance profiles were available for 4 of 6 patients. In adult patients, DTG monotherapy is associated with an increased risk of virological failure and DTG resistance [16]. Further, while dual therapy with DTG + 3TC is promising among adults, this study only included patients who were virologically suppressed at the time of switch [17]. Finally, while DTG-based ART is superior to LPV/r-based ART as a second-line therapy in the DAWNING trial, patients in this cohort were required to have at least 1 fully active NRTI [18]. Thus, for children who have high viral loads at the time of switch in combination with extensive drug resistance, the efficacy of DTG-based ART remains unknown. In this cohort of children, 4 of 6 patients had high viral loads at switch and at least one (patient 1) did not have any fully susceptible NRTI (although in addition to TAMs, M184 V was present which increases susceptibility to TDF). Thus in applying these findings to other cohorts of children, one must carefully consider the baseline NRTI resistance profile, as this may impact the long-term success of this regimen.

## Conclusions

In conclusion, this small size series indicates that children and adolescents may benefit from DTG- based regimen achieving a complete control of HIV infection with no side effects. The major advantages of DTG-based regimen are the possibility to reduce the pill burden to two pills once a day, the increasing treatment adherence and the low or absent risk of additional drug resistance mutations. Good safety profile was observed up to a maximum of 24 months in our patients.

## Abbreviation

3TC: Lamivudine
ABC: Abacavir
AIDS: Acquired immunodeficiency syndrome
ART: Anti-retroviral therapy
ATV: Atazanavir
CDC: Center for Disease Control and Prevention
D4T: Stavudine
DDI: Didanosine
DRV: Darunavir
DTG: Dolutegravir
ETR: Etravirine
FPV: Fosamprenavir
FTC: Emtricitabine
HIV: Human Immunodeficiency Virus
HR: High resistance
IDV: Indinavir
INSTIs: Integrase strand transfer inhibitors
IR: Intermediate resistance
LPV: Lopinavir
LPV/r: Lopinavir/ritonavir
LR: Low resistance
NFV: Nelfinavir
NVP: Nevirapine
RPV: Rilpivirine
RTV: Ritonavir
S: Sensible
SQV: Saquinavir
TDF: Tenofovir EFV Efavirenz
TPV: Tipranavir
ZDV: Zidovudine
